# An innovative care model for urgent and emergency care: A qualitative evaluation of community unscheduled care co-ordination hubs

**DOI:** 10.1371/journal.pone.0356237

**Published:** 2026-08-19

**Authors:** Suzanne Ablard, Jedidah Mould, Rosemarie Gough, Colin O’Keeffe, Maxine Kuczawski, Fiona Sampson, Natasha Treagust, Suzanne Mason

**Affiliations:** 1 The School of Medicine and Population Health, University of Sheffield, Sheffield, United Kingdom; 2 Faculty of Biology, Medicine and Health, School of Health Sciences, University of Manchester, Manchester, United Kingdom; 3 Critical Care, Sheffield Teaching Hospitals NHS Foundation Trust, Sheffield, United Kingdom; Universiti Sains Malaysia, MALAYSIA

## Abstract

**Background:**

High demand for emergency care continues to place strain on ambulance services and emergency departments. Many patients present with acute problems that, while requiring an urgent response, do not require hospital care. Providing safe alternatives to hospital conveyance for these patients is a priority. Community unscheduled care co-ordination hubs (UCCH) support clinicians (e.g., paramedics) to identify and refer patients with acute care needs into community services, allowing patient care close to or in their own home and reducing ambulance callouts or conveyance to hospital. Our study identified the barriers and enablers to the operation and impact of UCCHs in three locations across England.

**Methods:**

Twenty-one semi-structured interviews were conducted with clinical and non-clinical staff working in UCCHs, and clinical staff working in services which refer patients into UCCHs (e.g., paramedics). Transcripts were analysed thematically.

**Results:**

Four themes were identified: (1) UCCHs promote integrated community working by improving clinicians’ access to rapid community-based support for patients; (2) there was a tension between maintaining easy access for referring clinicians and ensuring consistent referral decision-making, with unclear eligibility criteria creating uncertainty about the remit of UCCHs (3) UCCH staff perceived limited ambulance service engagement as a key barrier to maximising the service’s impact on avoiding unnecessary hospital admissions; (4) Recruiting and retaining individuals with the right skills mix was a significant challenge, limiting the pace at which the UCCH could expand.

**Discussion:**

UCCHs have the potential to simplify access to community services and support avoidance of unnecessary ED conveyance. However, their impact may be limited by inconsistent referral processes, variable engagement from ambulance services, and workforce challenges. Addressing these factors may enhance the effectiveness, sustainability, and scalability of UCCHs as part of wider efforts to deliver urgent care closer to home.

## Background

Demand for hospital-based care is rising, leading to significant challenges in providing timely access to services [1]. In England, between 2023−24, 72.1% of patients attending emergency departments were seen, treated and discharged within four hours. This fell well below the national target of 95%, which has not been met since 2013−14 [2]. In addition, between 2018/19 and 2021/22 over one-fifth of patients conveyed to hospital by ambulance experienced handover delays exceeding 30 minutes, despite a target of 95% of handovers being completed within this timeframe [3]. These pressures indicate current levels of demand are unsustainable requiring the development of innovative solutions.

Previous research indicates that a significant proportion of patients attending EDs could be appropriately managed in alternative community-based settings. In response, the UK ambulance service have introduced strategies such as “hear and treat” and “see and treat” to reduce unnecessary conveyance to hospital. Currently, around a third of patients who receive an ambulance are not conveyed to the ED and are discharged at scene [4]. However, evidence of significant variation in the non-conveyance rates to hospital between ambulance services suggests potential for further improvement in these approaches [5].

One of the challenges in diverting patients to alternative community-based services is the fragmented nature of the health system. This lack of integration makes it difficult for both referring clinicians and patients to navigate and access appropriate support. Consequently, the ED is often used as a last resort, when other options are perceived as being too difficult to access [6].

To address these challenges, between 2019–2020 NHS England’s Emergency Care Improvement Support Team (ECIST), piloted unscheduled care co-ordination hubs (UCCHs). The aim of these hubs is to improve access to community-based services for patients with urgent but non-life-threatening healthcare needs, thereby reducing unnecessary conveyance to hospital. UCCHs provide a single point of access within the community and are staffed by multidisciplinary teams comprising senior clinical decision makers and non-clinical professionals. They accept referrals from ambulance services, primary care and other community services. The hubs fulfil several functions, including directing referrers to the most appropriate community-based service, or where required, delivering a community-based rapid response. In the latter case, senior clinicians (e.g., Advanced Clinical Practitioners) from the UCCH visit the patient in their own home within 2-hours to undertake a clinical assessment to determine whether further intervention is required. Essentially, this model enables referring clinicians to use a single access point to determine the most appropriate referral pathway for their patient, thereby making it easier to identify alternatives to ED conveyance.

The UK Governments recent 10-year health plan includes ambitious targets to integrate a range of professionals within patient-centred models of care, deliver more services closer to home, and reduce fragmentation across the system [6]. In this context, lessons can be drawn from the experiences of healthcare professionals involved in implementing UCCHs to help inform the delivery of these policy ambitions.

We undertook a qualitative evaluation of UCCHs in three sites in England to capture the perspectives of healthcare professionals working within UCCHs as well as clinicians who refer patients into the UCCH, identifying the challenges and successes they encounter, and opinions on how the UCCHs need to develop in the future.

## Methods

### Design and setting

We used an inductive qualitative design to conduct semi-structured interviews across three sites in England (United Kingdom) which had implemented a UCCH. Four UCCH sites were approached initially, but one UCCH site declined due to operational challenges restricting staff availability to participate in the interviews. UCCH sites were approached based on the site being established for at least 12 months, as this was considered a reasonable time for the sites to be set up and beginning to have impact on the UEC systems in which they operated. A national lead for the implementation of UCCHs across England acted as a gatekeeper to facilitate initial contact with service leads at each of the UCCHs.

**Fig 1:**
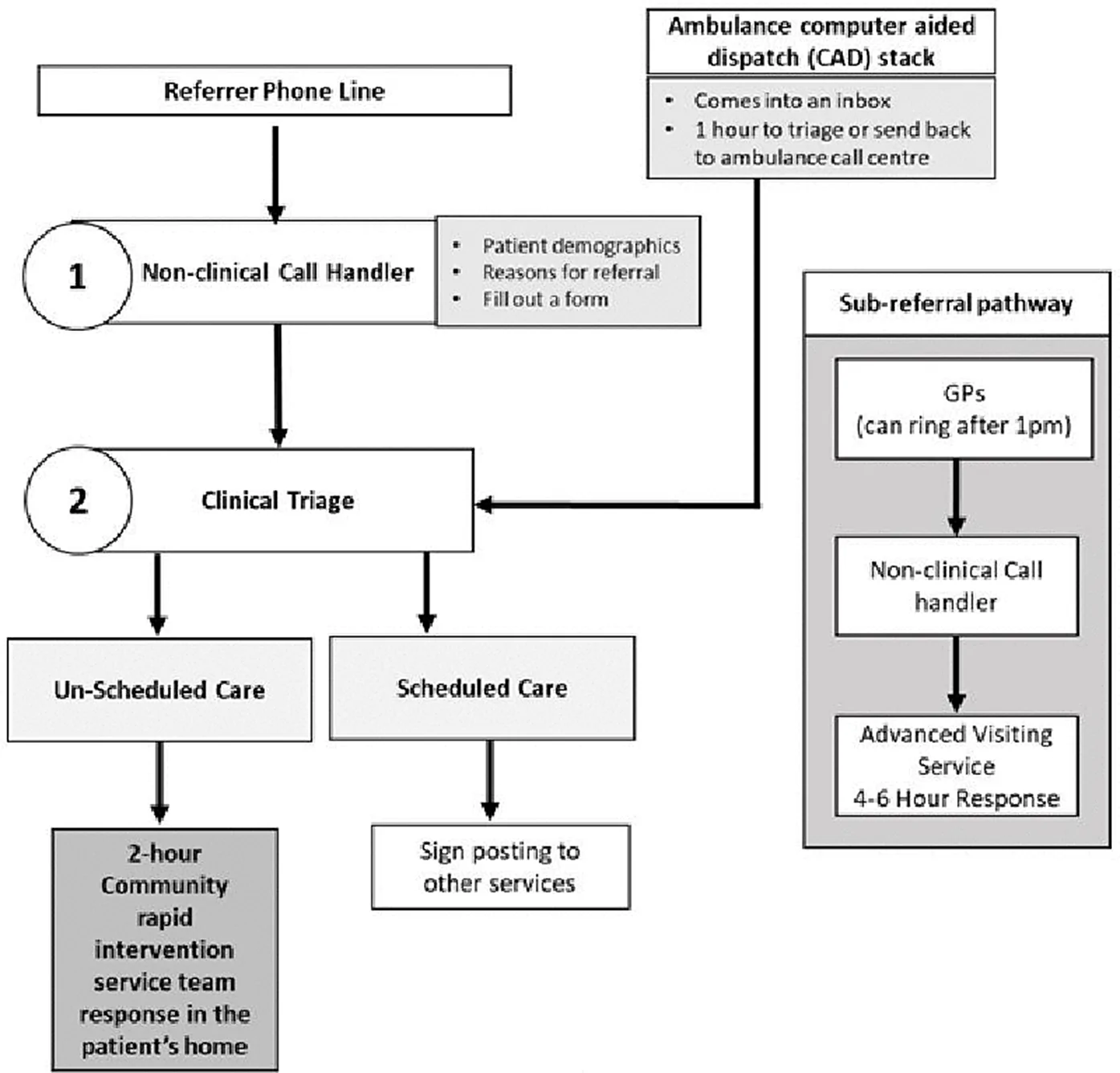
Diagram showing the referral process at site one.

**Fig 2:**
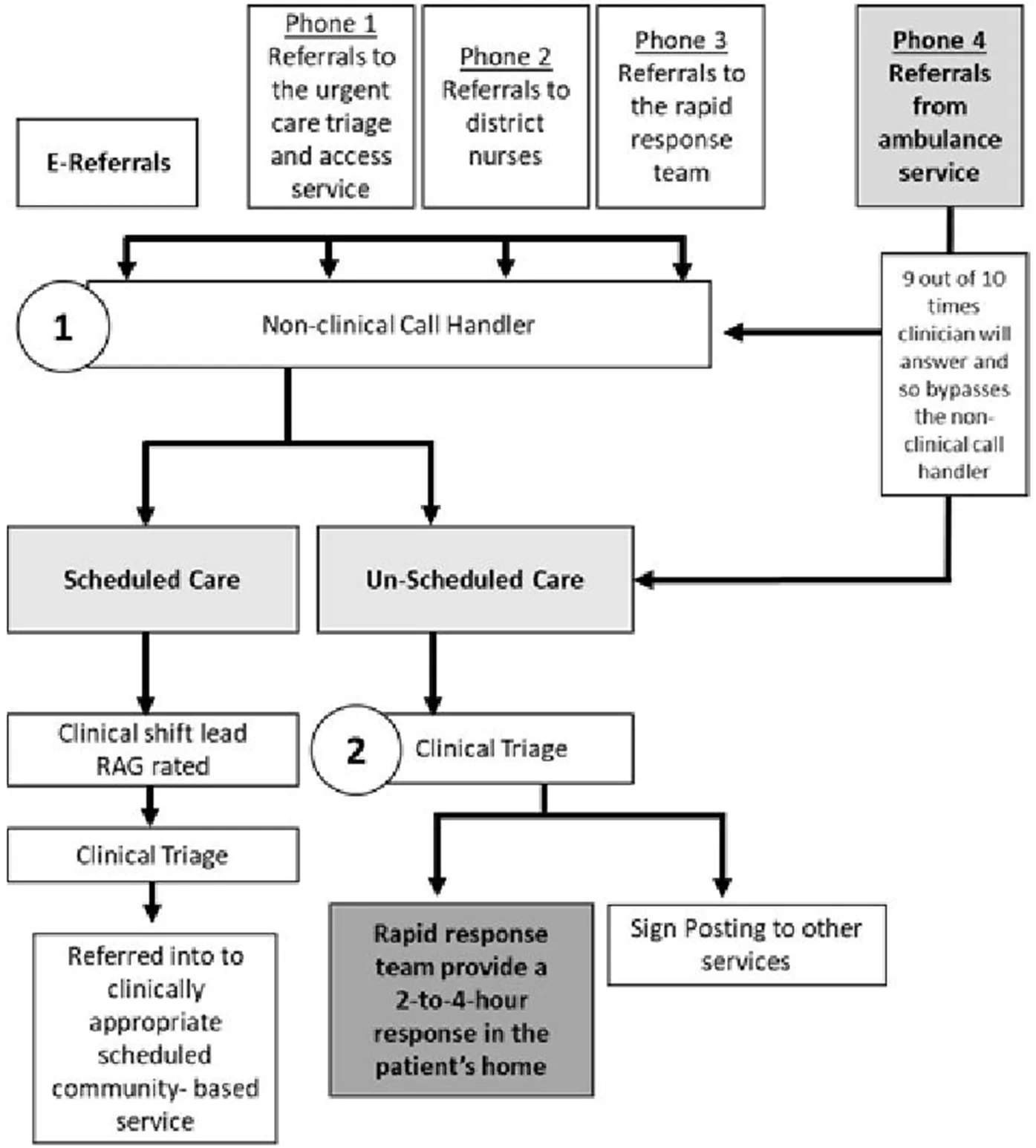
Diagram showing the referral process at site two.

**Fig 3:**
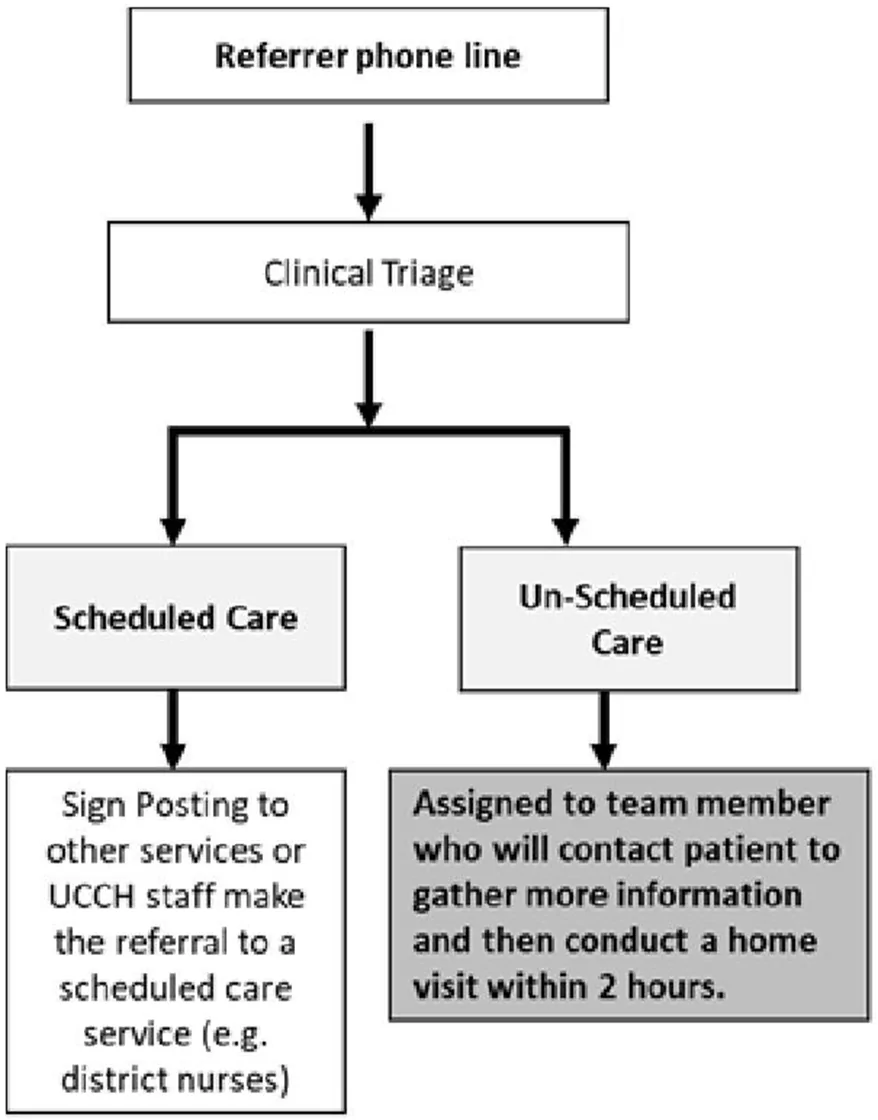
Diagram showing the referral process at site three.

Figs 1–3 provide a summary of the different UCCH models evaluated in this study.

Whilst the three UCCH models evaluated in our study shared a common overarching aim of improving access to community-based services to support alternatives to hospital conveyance, there were differences in their internal service structures. In particular, the services varied in the range of referrals accepted and the organisation of their call-handling processes.

Two sites (Fig 1 and Fig 3) accepted referrals only for patients with unscheduled healthcare needs. Referrals relating to scheduled care needs were signposted to alternative community services. In contrast, the third site (Fig 2) accepted referrals for both unscheduled and scheduled care needs.

In two sites (Fig 1 and Fig 2), calls were initially answered by a non-clinical call handler. In one of these sites (Fig 1) the non-clinical call handler collected basic referral information before transferring to a clinical call handler who triaged the patient and determined whether a rapid response was required or whether the referrer should be directed to a non-urgent community service. In the other site (Fig 2), the non-clinical call handler conducted an initial assessment to determine whether the referral related to scheduled or unscheduled care and directed the call to an appropriate clinician within the UCCH. In the third site (Fig 3), referrals were handled directly by a clinical call handler, rather than a non-clinical call handler.

In all three sites, they had a “rapid response team”. If after a clinician-to-clinician conversation it was determined that the patient had an unscheduled care need then the rapid response team (typically advanced clinical practitioners) could be dispatched to visit the patient at home within two hours. This provided referring clinicians, particularly the ambulance service, with a viable alternative to ED conveyance, ensuring patients received timely assessment and intervention at home while avoiding potentially unnecessary hospital attendance.

### Sampling and recruitment

We aimed to understand the experiences of three groups of people who either worked in or referred into the UCCH, as follows: (1) Clinical and non-clinical staff working in the UCCH; (2) service leads/ operational managers involved in the implementation and operation of the UCCH; (3) clinicians working in services which refer patients into the UCCH (e.g., paramedics, GPs).

In the first instance, the service lead at each UCCH site identified potential healthcare staff for interview based on the criteria above. Snowball sampling was then used to widen the pool of potential participants.

Eligible participants were provided with a study pack which included a cover letter, information sheet and consent form.

Recruitment took place between 12/05/2022 and 06/09/2022.

### Data collection

We designed a semi-structured interview guide, [see S1 and S2 Files] which was adapted for each participant group. Interviews with staff working in the UCCH explored: the participants role within the UCCH; why the UCCH was set up; their experience of working within the UCCH (including any barriers and facilitators to the operation of the UCCH); suggestions about how the UCCH could be developed in the future; and perceived impact on the Urgent and Emergency Care (UEC) system. Interviews with staff working in services which refer into the UCCH explored: their awareness of the UCCH and its purpose within the local UEC; their experience of referring patients into the UCCH (including barriers and facilitators); perceived impact of the UCCH on the UEC system; and suggestions about how the referral process into the UCCH could be improved in the future.

Interviews were conducted online (using Google Meet) or face to face by three authors (SA, JM, RG) who are experienced qualitative researchers with no clinical background or experience. This facilitated an independent perspective on UCCH operations and helped identify issues that may be overlooked by researchers embedded within the NHS. When participants used unfamiliar clinical terminology during the interview, the interviewers sought clarification to ensure they had an accurate understanding. In addition, one co-author (SM) has a clinical background and was consulted where necessary to support the interpretation of clinically related data.

Interviews were audio-recorded using an encrypted voice recorder. On completion of the interview, participants received a £30 shopping voucher to thank them for their time.

### Analysis

Interviews were transcribed verbatim and analysed using thematic analysis following the stages described by Braun and Clarke [7]. In the first stage, three of the authors (SA, JM, RG) independently coded a subset of the transcripts. The purpose was to familiarise themselves with the data and to develop an initial coding framework for analysing the remaining interviews. They then met to discuss and compare their coding and worked together to design the initial coding framework. Using this framework, the three authors proceeded to code the rest of the transcripts. However, the framework was used iteratively and refined throughout the analysis. Regular meetings were held to discuss emerging themes and update the coding framework as new insights arose. Two additional authors (FS and MK) did not participate directly in coding the transcripts but contributed to the analysis by reviewing emerging themes and suggesting further refinements. NVivo V.12.0, [8] was used to help structure the analysis.

### Ethical considerations

All participants received an information sheet to review before agreeing to take part in the study. Informed consent was then obtained through a signed written consent form prior to the interviews. The School of Medicine and Population Health Ethics Committee based at the University of Sheffield granted ethical approval for the study (Ref 045321).

## Results

We interviewed twenty-one people across three UCCHs: Sixteen worked in a UCCH (Twelve clinical and four non-clinical), and five referred patients into the UCCH (four paramedics and one GP). Table 1 provides an overview of the participants.

**Table 1:** Overview of the participants.

	Site 1	Site 2	Site 3
Healthcare staff working within the UCCH			
Management (e.g., service lead)	1	3	2
Clinical (e.g., triage nurse; advanced clinical practitioner)	1	3	2
Non-clinical (e.g., administrator)	0	4	0
Healthcare staff working in services which refer patients into the UCCH			
Ambulance service (e.g., paramedic)	2	1	1
Primary care (e.g., GP)	0	1	0
**TOTAL**			
	4	12	5

Interviews took place between 12/05/2022 and 06/09/2022. Interviews lasted between 35 minutes, and one hour forty-five minutes.

### Overarching themes

Table 2 provides an overview of the themes and sub-themes that were identified.

**Table 2:** Themes and subthemes.

Theme	Sub-theme
UCCHs facilitated integrated community working	• Before the UCCH, accessing community services was complex and time-consuming, primarily due to referring clinicians’ limited understanding of the scope and remit of different community services. • The UCCH has simplified this process by acting as a single point of access for unscheduled community care, improving access to community services in a timely manner.
Negotiating access to the UCCH rapid response team: uncertainty around the referral criteria and inconsistent acceptance decisions	• UCCHs had vague and non-specific referral criteria, focusing on “clinical conversations” with referrers to determine the best care for patients. • Some referring clinicians expressed frustration with inconsistent decision-making by UCCH staff regarding patient eligibility for rapid response services, leading to uncertainty about the UCCH’s remit. • UCCH staff described receiving referrals into the UCCH that they considered to be ‘inappropriate’ (i.e., cases UCCH staff believed were too unwell to be managed by the UCCH or conversely were more primary care type presentations). • Regular communication with referrers helped clarify referral guidelines.
The ability of the UCCH to maximise its impact on avoidable admissions was perceived to be dependent on ambulance service buy-in and engagement.	• UCCH staff saw strong potential to reduce urgent care demand by collaborating with ambulance services to improve access to community-based care, though support from ambulance teams varied across sites. • Trust-building between UCCH and ambulance staff was slow, hindered by communication issues, such as faulty phone systems, and previous negative referral experiences. • Low initial awareness of UCCH among paramedics improved through information campaigns and endorsement from senior ambulance managers, leading to increased referrals. • UCCH staff suggested that real-time access to the ambulance Computer Aided Dispatch (CAD) system could enable more proactive referrals (“pull model”), but governance concerns prevented this. Instead, a “push model” was trialled, where ambulance staff identified suitable patients for UCCH triage from the CAD stack. While this showed some success, it relied heavily on staff understanding of UCCH referral criteria.
Recruiting and retaining individuals with the right skills mix was a significant challenge, limiting the pace at which the UCCH could expand	• UCCHs adopted a multidisciplinary team model for their 2-hour urgent community response teams, fostering a supportive environment where diverse professionals worked together to provide holistic problem solving to patient care. • Recruitment and retention challenges hindered growth and impacted morale. This was mitigated by strong leadership and team building opportunities. • Senior UCCH managers were concerned that pressure from higher-level policies to quickly expand the service was unrealistic because ongoing recruitment and retention issues were limiting the resources needed to manage more patients.

### Theme one: UCCHs facilitated integrated community working

Prior to the implementation of the UCCH, accessing community services across the three sites was complex and time-consuming. Due to a lack of clarity regarding the scope of different services, referring clinicians (e.g., GPs, paramedics) often reported having to contact multiple services before being directed to the correct one. In these situations, it was sometimes easier and quicker to send patients directly to the hospital, rather than explore alternative pathways in the community.

*“I might say ‘I want a District Nurse’, but the District Nurse might say ‘well it’s not me, I can’t do this catheter, I don’t do catheters, it needs to be the Incontinence Team’. So we used to get this issue all the time where doctors would refer to the wrong person or not the ideal person, not the best contact first time.” [Referrer – Primary care]*

The UCCHs across all three sites aimed to simplify this process by creating a single point of access for unscheduled community care, with one site offering a single point of access for both unscheduled and scheduled community care. Participants explained that the main drivers of the UCCH were to make triage and referral across multiple community services easier, build collaboration among community and other UEC services and reduce unnecessary referrals to the emergency department by GPs, ambulance services, and care homes. One participant described the UCCH as:

*“Bridging the gap between all the different services and kind of working jointly together in an integrated way rather than us working in silos where we just do our own thing… so you can imagine, we’ve all got these different teams in adult community and then they’re all doing their own triage. They’re all doing their own allocation. Then you’d get a referral, and the patient might have needs that stem over multiple services… So that’s why we kind of pull all the triage and call handling into a central function.” [UCCH staff - Management]*

Referring clinicians valued the single point of access for unscheduled community care provided by the UCCH. For example, paramedics could refer patients into community services quickly, which is crucial in time pressured clinical environments.

“*We’re dead lucky how the team handle it, you just ring them up and literally within 20 minutes between the team having a discussion it’s been sorted and nine times out of ten, they’re normally left at home.” [Referrer – Ambulance service]*

*“For an ambulance paramedic, a single point of access is absolutely the way to go because it just takes away all of that complication, it saves time. And they don’t have, essentially paramedics don’t have that time to waste because the patient might not be dying but that paramedic needs to wrap it up kind of thing, because they need to go to the next emergency call.” [Referrer – Ambulance service]*

### Theme two: Negotiating access to the UCCH rapid response team: uncertainty around the referral criteria and inconsistent acceptance decisions

A key challenge for UCCH staff and referring clinicians was identifying patients whose health care needs were urgent enough to warrant a rapid response, but who could still be safely managed at home. Determining the most appropriate pathway for these patients was often not straightforward, as many fell into a grey area between routine community care and hospital conveyance. To accommodate this clinical uncertainty and reduce barriers to access, none of the UCCHs had strict referral criteria. Instead, any eligibility criteria they had tended to be broad and non-specific, with a stronger emphasis placed on having a “clinical conversation” between the UCCH and referrer to determine the best place of care for the patient. This allowed referral decisions to be made on a case-by-case basis, drawing on the clinical judgement of both parties and supporting shared responsibility for managing risk. UCCH staff felt this flexible approach encouraged clinicians to seek advice and explore community-based alternatives to hospital conveyance, particularly in cases where the most appropriate course of action was unclear.

*“…encourage a clinical conversation, because we would rather them have a conversation and speak to care co-ordination centre than think, oh, maybe not, not too sure, I’ll just take them to hospital.” [Referrer – Ambulance service]*

This approach was viewed as preferable to a narrowly defined referral criteria, which participants felt could create barriers to access. When eligibility criteria were perceived as overly restrictive, clinicians could become reluctant to refer patients and may stop using the service altogether, as one paramedic noted:

*“So we had one similar but it was run by district nurses and I’m sure with that service if you sneezed three times and you couldn’t wash the dishes then they wouldn’t accept you. It was ridiculous…It got to the point where we just didn’t use it and they were saying ‘oh we’re having no referrals from the Ambulance Service and this is your criteria’ and the criteria was like if you’d broken a nail you can’t do it, it was ridiculous so we just stopped using it so eventually it got shut.” [Referrer – Ambulance service]*

Furthermore, UCCHs reported using a “no wrong door” policy. If after a clinical conversation with the referrer it was determined that a patient did not require an unscheduled community response (e.g., rapid 2-hour response), UCCH staff would signpost the referrer to the appropriate alternative community service or make the referral on their behalf. This approach encouraged referring clinicians to reach out, even if they were unsure of the patient’s exact needs.

*“So it’s a lot of signposting if it isn’t us rather than just a blanket ‘no’. We always try and help and we’re a good team to know what other services might help.” [UCCH staff - Clinical]*

However, due to the absence of strict referral criteria, decisions about whether a patient received a rapid community response or were signposted to an alternative service ultimately depended on the clinical judgement of the individual UCCH staff member handling the referral. Some referrers felt this resulted in inconsistent decision making, with similar patients accepted on one occasion but not another. This perceived variability in referral outcomes created uncertainty about the UCCH’s remit in accepting patients for a rapid response.

*“My main bug bear, I don’t know if that’s the right word, is it depends who you speak to on the phone, which is I think they lack consistency” [Referrer – Ambulance service]*

*“There can be frustration about, well depending on who’s on the referral line depends on whether that case gets accepted or not… So I think there is work for us to do and there’s feedback around that.” [UCCH staff - Management]*

Conversely, UCCH staff raised concerns about the appropriateness of referrals received from referring services, including primary care, ambulance services, and care homes. For example, some patients assessed and referred by organisations such as the ambulance service were left at home with follow-up care from the UCCH’s 2-hour urgent community response team. However, when the response team arrived at the patients’ homes, they judged that the patient was at too high a risk for home care and should not have been left at home in the first place. UCCH staff also reported instances where clinicians appeared to exaggerate symptoms or use “trigger words” to meet the referral thresholds for the UCCH’s 2-hour urgent community response team, often in the context of wider system pressures and limited capacity.

*“So I think that sometimes people, sometimes they’ll use the rapid intervention team as a bit of a mop up service if I’m honest. I think it’s abused sometimes when people know the trigger words of what to say to get somebody to come out to them… but you know if everybody’s just under so many pressures aren’t they” [UCCH staff - Clinical]*

UCCH staff also described receiving frequent referrals from inexperienced agency staff employed in care homes who were seeking reassurance and advice about how to deal with low acuity issues (e.g., catheter problems). A solution piloted by one UCCH involved providing online educational sessions to care home staff to reduce the number of inappropriate primary care type presentations being received.

*“They’ve recently, the last month or so, they’ve got a twelve-month agenda, of every month one of the ANPs on Teams provides some basic teaching to their level of like what to look for. Because a big part of what the care homes ring us in with are UTIs, chest infections… or catheter problems” [UCCH staff – management]*

Overall, UCCHs faced ongoing challenges in balancing flexible referral processes that improved accessibility with the need for consistency in decision making Regular communication and feedback between the UCCH and the ambulance service was reported to have some success in improving mutual understanding and clarifying which patients were appropriate for a 2-hour urgent community response.

*“I have constant engagement with the care co-ordination centre, two times a week. And we have an opportunity to, to talk through referrals that have not been accepted and understand why those referrals have not been accepted. And then from that we can take learning to kind of map out what it, what they will take. So it’s a constant ever learning environment.” [Referrer – Ambulance service]*

### Theme three: The ability of the UCCH to maximise its impact on avoidable admissions was perceived to be dependent on ambulance service buy-in and engagement

UCCH staff believed that the ambulance service represented a key source of patients who might otherwise be conveyed to the hospital. Consequently, increasing referrals from the ambulance service was viewed as important to help maximise the UCCH’s impact on admission avoidance. However, achieving this required buy-in at both managerial and frontline levels within the ambulance service. However, UCCH staff described varying levels of agreement between the UCCH and ambulance service regarding the scale of opportunity for the UCCH to support admission avoidance efforts.

*“So they very much believe that every patient they take up is appropriate. And we do have a low conveyance rate to be fair…There was a big argument over what the scale of opportunity was. And they wanted everything to prove that they were right… But the reality is strategic level block… we need them to play ball and to engage in order to get our ducks in line to be able to offer what we think, you know, would work.” [UCCH staff - management]*

*“Getting that senior support at exec level for what we’re trying to do and them understanding the vision that’s been really helpful and being able to showcase what we’re doing and the benefits of that.” [UCCH staff - management]*

There was evidence of a cascading effect, whereby support from senior ambulance service leaders positively influenced engagement among frontline paramedics. Specifically, a lack of awareness of the UCCH among paramedics was believed to contribute to the overall low number of referrals from the ambulance service. However, where strategic leaders recognised the value of the UCCH, they actively promoted it’s use through top-down messaging and organisational support, increasing awareness and encouraging greater utilisation by frontline staff.

*“They are promoting them every day saying ‘you can go up to A&E and sit there the rest of your shift or ring the CRIS team’ … ‘Oh the boss has told me I can do it, let’s ring them and find out’…” [Referrer – Ambulance service]*

Building trust and confidence in the UCCH was described as a slow, and challenging process, and could be undermined quickly by negative experiences. A paramedic explained that a negative referral experience with the UCCH could quickly spread among crew members, reducing confidence in the service and discouraging future referrals. For example, one UCCH, experienced a significant decline in referrals following a temporary telephone system failure that made the service difficult to contact. Even though the phone issue was resolved quickly, it took considerably longer to rebuild paramedics’ trust and increase the referrals again. Highlighting how easily confidence in the service could be lost and how difficult it was to regain

*“We had a rapid launch week where we received, I think, 87 calls in that one week but then we had an issue with our phones. We had a system upgrade overnight which made the phones fail for the next day. It took a few days to get them back on properly and the paramedics just lost trust.” [UCCH staff - management]*

Another paramedic mentioned hearing about the success of electronic “feedback loops” as an effective way to encourage the ambulance service to use new health service interventions. An example of a feedback loop is when a paramedic refers a patient to the UCCH, and the UCCH then provides feedback on the outcome of the referral – such as the patient avoiding a hospital conveyance. In theory, hearing about the positive outcome would lead the paramedic to using the service again in the future. However, this approach had not been adopted by any of the UCCHs evaluated in this study.

*“I know one of my counterparts, a paramedic called [name], she set up a really good feedback loop… the crews would get the e-mail to say, this is what happened, you know, we’ve put these interventions and stuff like that, just a little bit of learning, and what, with that feedback I think her referrals increased… it stopped and then everything kind of decreased again.” [Referrer – Ambulance service]*

UCCH staff felt that greater integration with ambulance service workflows could substantially increase the UCCHs reach and impact of the UCCH. For example, some UCCH staff believed that having direct access to the ambulance caseload in real time via the 999 computer-aided dispatch (CAD) system (‘pull model’), would enable UCCH staff to proactively identify suitable patients and transfer them into community-based care before an ambulance was dispatched. UCCH staff believed this could increase referrals and reduce unnecessary ambulance conveyances. However, senior managers within the ambulance service raised concerns about granting the UCCH access to the CAD system, citing governance challenges and the ambulance service’s responsibility for overseeing patients in their CAD stack, limiting progress towards implementation.

An alternative approach being trialled in some UCCHs involved ambulance Emergency Operations Centre staff identifying suitable category 3 and 4 patients and referring them to the UCCH for triage by UCCH staff (‘push model’). These pilot exercises showed some success but still relied on the ambulance service being aware of the UCCH and its criteria for accepting patients.

*“We went and had a look at the stack when this was all first mooted and in a 2-hour period there were 9 fallers on the floor, 8 of whom we felt would have been suitable for our service. And when we left 4 hours later, they were still on the floor. You know, that’s that’s quite sobering isn’t it?” [UCCH staff – clinical]*

*“There’s been a lot of resistance from our ambulance service. We were trying to, with [Name]’s support, about 18 months or two years ago to get in and look at the ambulance CAD stack and wanting to pull patients. But they have been very very resistant… put in an immense amount of effort into trying to get things going and they didn’t get off the ground because of engagement” [UCCH staff – management]*

### Theme four: Recruiting and retaining individuals with the right skills mix was a significant challenge, limiting the pace at which the UCCH could expand

All UCCHs implemented a multidisciplinary team model within their ‘2-hour urgent community response team’, recruiting paramedics, social workers, nurses, physiotherapists, and occupational therapists. UCCH staff expressed enjoyment in working with colleagues from diverse professional backgrounds, describing the environment as supportive and appreciating the wide range of expertise brought by different team members. This diversity allowed for more holistic problem-solving, as each team member’s unique expertise addressed various aspects of patient care.

*“…I think in our team we’re really lucky because we’ve got a lot of people from different areas…there’s always somebody at the end of the phone there who’s got a wealth of experience in emergency situations and advice for anything that you’re worried about…we’ve got a good support for clinical sort of problems that we might face” [UCCH staff – clinical]*

While multidisciplinary working was widely viewed as a strength of the UCCH model, staff in one UCCH model described challenges when professional boundaries became less clearly defined. To increase workforce flexibility, the service attempted to upskill all members of the multidisciplinary team to be able to assess, diagnose, and treat patients to the same level regardless of professional background. While this model initially worked, some professional groups, such as occupational therapists, felt they lacked the necessary clinical expertise to manage the most acutely unwell patients. This led to a significant decline in staff morale. In response, senior management within the UCCH began re-evaluating the workforce model to better align the skills of individual professional groups with the specific needs of patient care.

*“The model that we run in terms of upskilling everyone to provide a standard assessment is fine until the patients start to get really sick. And then you’re sending out an occupational therapist to a really sick patient that’s really undifferentiated… So an occupational therapist will be upskilled to take obs and read them but… their professional background’s not to interpret obs or to come to any medical diagnosis …there is professional groups that feel unhappy with the risk that they individually carry with their professional background.” [UCCH staff – Management]*

UCCH staff described recruitment of roles within the UCCH as challenging because the role required a combination of skills not commonly found within a single professional background. Due to the complexity of delivering acute-level care in patient’s homes, UCCH staff felt clinical roles required knowledge of community-based health services, as well as experience of managing acutely unwell patients. Recruiting individuals with both skill sets was difficult, leading UCCHs to develop in-house training programmes to address knowledge gaps. However, these programmes were described as time consuming and took staff a long time to become fully competent in the role,

*“Trying to get the right people with the right skills is very difficult… I don’t think you’ll ever get a perfect person with the right skill set. I think you need somebody who knows when someone’s not very well and needs hospital… I think they do need to have acute experience because once you’ve looked after a sick patient then you recognise a sick patient.” [UCCH staff - manager]*

*“I think that’s very important for care co-ordination to have members of staff with that community background, because if you speak to nurses from the acute, they don’t know what happens in the community.” [UCCH staff – clinical]*

Retention issues were also reported across both clinical and non-clinical roles, although the reasons varied between staff groups. For example, retention of triage nurses in the UCCH was difficult because it is not a patient-facing role. In one UCCH, triage nurses who couldn’t deliver face-to-face care during the COVID-19 pandemic had been re-deployed to work in the UCCH, as one person said:

*“We had to do it because we were shielding from COVID because of our health issues. So we had no choice really so then we adapt.” [UCCH staff - clinical]*

While this non-patient-facing role initially helped set up the service, misunderstandings about role specifications and a desire for face-to-face patient contact led to challenges in recruiting and retaining more triage nurses. Senior UCCH staff were discussing ways to incorporate face-to-face patient contact into the triage role, but this would first require an increase in the number of triage nurses recruited to the UCCH.

*“So, care coordination is primarily a bit of an office-y desk job. However, our vision is for it to be a little bit of both… we wanna get out there to patients that don’t fit a particular box… So we’re doing a bit of a pilot around stuff like that which might then aid us retaining some of our staff … but again you need the right infrastructure around you to support you to do these things because your work streams in the office can’t fall because we’re going out to do face to face contact. So, we’ve got to get a balance.” [UCCH staff – Management]*

In one UCCH, there were challenges retaining non-clinical administrative staff. Administrative staff mentioned that many viewed the role as a stepping stone into the NHS, often moving on to more senior positions in other departments in pursuit of career progression.

*“I’ve been here for five years and it’s like a constant turnaround of staff … the majority are using it like a stepping stone into the NHS and they’re moving on quite quickly … to develop their careers… I mean nothing wrong with that… but then it’s constant lack of staff.” [UCCH staff – non-clinical]*

Recruitment and retention challenges negatively impacted staff morale, but strong leadership helped mitigate this effect. In one UCCH, staff morale improved following the introduction of team-building exercises and away days, which provided an opportunity for staff to connect and support each other in a less formal setting.

*“I think the team building because obviously that’s important and we, you don’t get much when you’re triaging and having conversations quite regular throughout the day. We’re having away days as well. It’s like lunches, like a couple of hours where we go and have something to eat and a catch up and we’ll get time to, to talk to each other…. That’s quite nice, that’s something new as well.” [UCCH staff – clinical]*

Senior management within the UCCH expressed concern about the pressure from top-down policy and commissioning requirements. Constraints within the UEC system created pressure to rapidly expand the UCCH service, as it was seen as a potential solution for reducing hospital conveyances. However, senior managers felt the timelines for these changes were unrealistic, given ongoing recruitment and retention challenges within the UCCH, which limited available resources to manage an increased patient case load.

*“I just think there’s so many demands on us at the moment to grow the service…there’s lots of initiatives coming in and you know I’ll be honest as a manager I’m feeling very overwhelmed …So we have ambulances queuing outside [Hospital Name] and it’s not unusual for them to wait you know, for it to be a 12 hour. I mean last week there were 18 hour waits outside… So our services are being asked to scope and what extra we can put in and it just feels so unrealistic… you know I think our services are very well suited to try and support some of these initiatives but it feels very unrealistic in terms of time frames” [UCCH staff – management]*

## Discussion

We undertook a qualitative evaluation of UCCHs, which aim to make it easier for clinicians to access community-based services for patients with unscheduled care needs, helping to avoid unnecessary conveyance to the ED. Our study highlighted both the barriers and enablers in delivering these services. Specifically, we identified four key themes: (1) UCCHs promote integrated community working, improving clinicians’ access to rapid community support for patients; (2) there is a tension between maintaining easy access to the UCCH for referring clinicians and ensuring consistency in referral decision-making; (3) The ability of the UCCH to maximise its impact on avoidable admissions was perceived to be dependent on ambulance service buy-in and engagement; (4) Recruiting and retaining individuals with the right skills mix was a significant challenge, limiting the pace at which the UCCH could expand.

### Facilitation of integrated working

Before the introduction of UCCHs, community services operated in silos, each with its own triage and referral criteria. This made it difficult for referring clinicians to navigate the system, as they often lacked a comprehensive understanding of the available community services. As a result, clinicians frequently found themselves sent back and forth between different services before ultimately identifying the most appropriate option for their patients. Research indicates that this uncertainty can lead clinicians to rely on the ED as the most reliable option, even when hospitalisation may not be necessary [9].

The introduction of UCCHs addressed these challenges through a single point of access and a ‘no wrong door’ policy. Referring clinicians now only needed to contact one number, where they could engage in clinician-to-clinician conversations to determine the most appropriate community service. This streamlined access to community services significantly reduced the time clinicians spent navigating between providers to find the right service for their patient’s needs. Interviews highlighted that this approach was especially beneficial for paramedics, where minimising on-scene time is a top priority.

### Inconsistencies in referral practice

UCCHs operated on the principle of joint decision-making between UCCH triage staff and referring clinicians, with no strict referral criteria formally outlined, to promote ease of access and encourage use of the service by referring clinicians. This collaborative approach supports clinician decision-making about which patients could safely remain at home, increasing their confidence and reducing unnecessary hospital conveyances.

For example, research has shown that whilst paramedics are generally effective at identifying patients who need to be transported to hospital, they are often less confident in recognising those who could benefit from alternative care options [10]. This highlights the role of services such as the UCCH in supporting decision-making in situations of clinical uncertainty. Equally, paramedics with extended skills have been shown to have greater confidence in discharging patients at scene than paramedics with standard training [11]. The UCCH may help address these gaps in confidence by providing access to specialist advise and community-based alternatives to hospital care. Specifically, paramedics interviewed in our study praised the UCCHs for enabling them to safely leave patients at home, knowing that community services would provide the necessary wraparound care. However, the absence of formal referral criteria introduced variability in decision-making between UCCH staff members handling referrals. Referring clinicians in our study reported challenges related to inconsistent triage decisions, with similar patients sometimes receiving different outcomes depending on who they spoke to. This variability made it challenging for clinicians to understand which patients were appropriate for referral to the rapid response service.

Clearer referral criteria may improve consistency in decision-making. However, our findings indicate that there is a trade-off between consistency and accessibility, as overly prescriptive criteria may make it more difficult for clinicians to access the service, particularly for patients with complex or uncertain presentations.

In the future, there may be value in developing clearer referral criteria for the UCCH. However, it is essential to strike a balance, ensuring that referral criteria are not so restrictive that they limit access to the service. These findings highlight the ongoing challenge of defining and identifying patients within the “grey zone” of urgent but non–life-threatening need and determining who can be safely managed at home rather than conveyed to hospital.

### A shared vision and engagement between UCCHs and the ambulance sector

Increasing the number of referrals from the ambulance service was seen by UCCH staff as a key priority. Previous research has highlighted that high volumes of patients are conveyed to the ED with non-urgent health problems which could be managed in alternative healthcare settings [12]. One contributing factor is uncertainty around the safety of leaving patients at home, which can lead to more risk-averse decision-making [11]. The UCCH offers a potential solution to this by providing a two-hour community response, enabling ambulance teams to safely leave patients at home with appropriate community support in place. However, despite these potential benefits, UCCH staff felt that the ambulance service was underutilising the service.

One issue identified was a disconnect between the ambulance services and UCCHs perceptions around the potential scale of impact UCCHs could have in reducing hospital conveyances, an issue also highlighted in previous research [11]. Blodgett et al. (2017) highlighted that a successful paramedic pathfinder tool, which helped paramedics triage patients into categories such as ED conveyance, urgent care centre referral, GP referral, or self-care at home, worked due to paramedic enthusiasm and a shared vision for the service [13]. Unfortunately, this shared vision and buy-in was not always present in the UCCHs evaluated in this study. Furthermore, paramedics were often unaware of the UCCHs, and while trust in the service developed slowly, it was quickly lost when issues arose.

UCCH staff expressed a desire for access to the ambulance service’s CAD stack, as this would provide them with more control over identifying patients who could be safely managed in the community through a “pull model”. This would reduce their reliance on other services to refer patients into the UCCH. However, senior managers within the ambulance service were hesitant, citing concerns over governance and the ambulance services responsibility for overseeing patients in the CAD stack. The challenge of data sharing within the NHS extends far beyond the scope of UCCHs. Interoperability issues – the ability of different systems, organisations, and individuals to share and use information effectively – are unlikely to be resolved in the short term [14,15].

### Workforce challenges

Recruitment and retention of staff within the UCCHs emerged as a significant challenge across all the UCCHs evaluated in this study. While these workforce pressures reflect wider challenges experienced across the NHS [16], participants identified several challenges specific to the UCCH model. For example, UCCHs require individuals who not only possess the skills necessary for managing high-acuity patients but are also capable of handling the risks involved in caring for acutely unwell patients in their own homes. Finding the right skill mix to meet these requirements was challenging. As a result, UCCHs relied on in-house training programmes to address knowledge gaps and support staff in developing the required competencies. However, UCCH staff reported that it took considerable time to build the necessary knowledge and experience. Overall, workforce constraints within the UCCHs reduced service capacity and were perceived by managers a barrier to expanding UCCH services at the pace expected by national UEC priorities.

### Moving care closer to home

Overall, whilst our study was conducted in the context of UCCHs, the findings have broader applicability, particularly in relation to the UK Government’s ambition to deliver more care closer to home, as outlined in the NHS Long Term Plan [6]. These policy directions increasingly emphasise community-based alternatives to hospital care.

Our findings suggest that while UCCH-type services may support this ambition, implementation is not straightforward. Specifically, uncertainty remains regarding which patients can be safely managed in the community as an alternative to hospital admission, and decision-making in this area is not easily standardised.

Furthermore, safely delivering hospital-level care in patients’ homes requires a workforce who have the appropriate skill mix. Developing and maintaining such a workforce is challenging and time consuming, constraining the pace at which new models of care can be introduced.

### Limitations

We aimed to recruit staff working within UCCHs, as well as clinicians referring patients into these services. While recruitment of UCCH staff was successful, we encountered challenges recruiting referring clinicians, resulting in a smaller sample size for this group than originally planned. Recruitment of referring clinicians relied on UCCH staff identifying eligible participants. However, UCCH staff often had limited ongoing contact with these clinicians, who may only make occasional or one-off referrals.

In addition, non-clinical UCCH staff were recruited from only one case study site. Early interviews at this site indicated that clinical staff were able to provide particularly rich insights into the implementation and operation of the UCCH, including aspects not always visible to non-clinical staff. In response, and due to time and resource constraints a decision was made to prioritise recruitment of comparable clinical and managerial staff across subsequent case study sites to ensure sufficient depth of data to address the study’s research questions. Future research could consider including a broader range of staff roles than those represented in this study.

Furthermore, whilst participants discussed the potential impact of UCCHs on addressing demand within the UEC system, these insights are based on their personal perceptions. To gain a more comprehensive understanding of the actual impact UCCHs are having on addressing UEC demand, a follow-up quantitative study should be conducted. This would provide more objective data to assess the effectiveness of UCCHs in meeting the needs of the UEC system.

## Conclusion

UCCHs have the potential to simplify access to community-based services and support the avoidance of unnecessary ED conveyances. However, our study identified several factors that may limit their impact. Inconsistencies in decision-making regarding which patients are suitable for a rapid community response as an alternative to ED attendance created uncertainty among referring clinicians about service eligibility criteria. In addition, underutilisation and limited buy-in from the ambulance service contributed to perceptions among UCCH staff that the service was not operating at its full potential. Finally, ongoing challenges in recruiting and retaining staff with the appropriate skill mix constrained the ability of UCCHs to expand in response to increasing pressure from national policy directives.

## Supporting information

S1 File Supplementary information 1. (DOCX)

S2 File Supplementary information 2. (DOCX)

S3 File SRQR reporting guideline. (DOCX)
