# Supplementary material for: An innovative care model for urgent and emergency care: A qualitative evaluation of community unscheduled care co-ordination hubs
Source: PLoS One. 2026 Aug 19;21(8):e0356237. doi: 10.1371/journal.pone.0356237 (PMC13489415; doi:10.1371/journal.pone.0356237)
Supplement: Supplementary file 1 [file pone.0356237.s001.docx]

**Evaluation of Community Unscheduled Care Coordination to reduce hospital attendance**

**Topic Guide – Care Hub Staff**

**1. Can you start off by describing your role within the Care Co-ordination hub**

**2. Can you describe why the care co-ordination hub was set up (in location)?**

Prompts:

- What were the local drivers for setting up the care co-ordination hub model? (e.g system demand, capacity)

- What was the care co-ordination hub trying to achieve/address?

- Has it achieved these aims?

**3. I’d now like to ask you about how well the care hub model is working in [Location] – what is working well and what is not working so well?**

Prompts:

- What has worked well?

- What has helped this working well?

- What has not worked well?

- What has prevented this working well?

- Any unforeseen consequences? (what causes them?)

**4. Is there anything which could be improved with regards to how the care co-ordination hub operates on a day-to-day basis?**

**5. Next, I would like to hear your thoughts on what impact the care co-ordination hub has had on the Urgent and Emergency Care system, as well as wider NHS services**

Probe:

- What facilitates the positive impact on system?

- What prevents the hub impacting on the system?

**6. Please can you describe how you see the care co-ordination hub developing in the future**

Probe:

Do you foresee any challenges or barriers associated with expanding the care co-ordination hub in the future?

**7. Please can you describe what impact the care co-ordination hub has had on patient care**

**8. Finally, are there any other barriers, facilitators, or challenges which you would like to discuss?**
