# Supplementary material for: An innovative care model for urgent and emergency care: A qualitative evaluation of community unscheduled care co-ordination hubs
Source: PLoS One. 2026 Aug 19;21(8):e0356237. doi: 10.1371/journal.pone.0356237 (PMC13489415; doi:10.1371/journal.pone.0356237)
Supplement: Supplementary file 2 [file pone.0356237.s002.docx]

**Evaluation of Community Unscheduled Care Coordination to reduce hospital attendance**

**Topic Guide – Healthcare staff referring into the UCCH**

**1. Can you briefly describe your role within the [name service]?**

**2. Are you aware of the care co-ordination hub in [location] and its purpose /role in the local unscheduled care system?**

**3. Can you describe how the [name of service] began working with the Care Hub in [locality]?**

Prompts: Initial contact and set up of relationship, development over time, who from [service] is involved

**4. How successfully is the [name of service] working with the care hub in [locality]?**

**5. How has the Care Hub impacted on [name of service] demand & provision of care in (locality)?**

Prompts: Reducing unnecessary transfers to hospital in patients who have unscheduled care needs but are not seriously ill? Any unintended consequences?

**6. What needs to be in place to improve the way [name of service] works with the care hub in future**

**8. Have you received any feedback from patients who have been referred to the care co-ordination hub?**

Prompts : Patient expectations? Patient safety concerns?

**8. Is there anything else you would like to discuss?**
