# Supplementary material for: An innovative care model for urgent and emergency care: A qualitative evaluation of community unscheduled care co-ordination hubs
Source: PLoS One. 2026 Aug 19;21(8):e0356237. doi: 10.1371/journal.pone.0356237 (PMC13489415; doi:10.1371/journal.pone.0356237)
Supplement: Supplementary file 3 [file pone.0356237.s003.docx]

**Standards for reporting qualitative research (SRQR)**

**Title and abstract**

| **Title:** Concise description of the nature and topic of the study identifying the study as qualitative or indicating the approach (e.g. ethnography, grounded theory) or data collection methods (e.g. interview, focus group) is recommended. | File: Manuscript_FINALv1  Page: 1 |
| --- | --- |
| **Abstract:** Summary of key elements of the study using the abstract format of the intended publication; typically includes background, purpose, methods, results, and conclusions. | File: Manuscript_FINALv1  Pages: 2-3 |

**Introduction**

| **Problem formulation:** Description and significance of the problem/phenomenon; review of relevant theory and empirical work; problem statement. | File: Manuscript_FINALv1  Pages: 4-5 |
| --- | --- |
| **Purpose of research question:** Purpose of the study and specific objectives or questions. | File: Manuscript_FINALv1  Pages: 4-5 |

**Methods**

| **Qualitative approach and research paradigm:** Qualitative approach (e.g. ethnography, grounded theory, phenomenology, narrative research) and guiding theory if appropriate; identifying the research paradigm (e.g. postpositivist, constructivist/interpretivist) is also recommended; rationale. | File: Manuscript_FINALv1  Page: 5 |
| --- | --- |
| **Researcher characteristic and reflexivity:** Researchers characteristics that may influence the research, including personal attributes, qualifications / experience, relationship with participants, assumptions, and/or presuppositions; potential or actual interaction between researchers characteristics and the research questions, approach, methods, results, and/or transferability. | File: Manuscript_FINALv1  Page: 8 |
| **Context:** Setting/site and salient contextual factors; rationale. | File: Manuscript_FINALv1  Pages: 5-6 |
| **Sampling strategy:** How and why research participants, documents, or events were selected; criteria for deciding when no further sampling was necessary (e.g. sampling saturation); rationale. | File: Manuscript_FINALv1  Page: 7 |
| **Ethical issues pertaining to human subjects:** Documentation of approval by an appropriate ethics review board and participant consent, or explanation for lack therof; other confidentiality and data security issues. | File: Manuscript_FINALv1  Page: 8 |
| **Data collection methods:** Types of data collected; details of data collection procedures including (as appropriate) start and stop dates of data collection and analysis, iterative process, triangulation of sources / methods, and modification of procedures in response to evolving study findings; rationale. | File: Manuscript_FINALv1  Pages: 7-8 |
| **Data collection instruments and technologies:** Description of instruments (e.g. interview guides, questionnaires) and devices (e.g. audio recorders) used for data collection; if/how the instrument(s) changed over the course of the study. | File: Manuscript_FINALv1  Pages: 7-8 |
| **Units of study:** Number and relevant characteristics of participants, documents, or events included in the study; level of participation (could be reported in results). | File: Manuscript_FINALv1  Page: 9 |
| **Data processing:** Methods for processing data prior to and during analysis, including transcription, data entry, data management and security, verification of data integrity, data coding, and anonymization / de-identification of excerpts. | File: Manuscript_FINALv1  Page: 8 |
| **Data analysis:** Process by which inferences, themes, etc., were identified and developed, including the researchers involved in data analysis; usually references a specific paradigm or approach; rationale. | File: Manuscript_FINALv1  Page: 8 |
| **Techniques to enhance trustworthiness:** Techniques to enhance trustworthiness and credibility of data analysis (e.g. member checking, audit trail, triangulation); rationale. | File: Manuscript_FINALv1  Page: 8 |

**Results / findings**

| **Synthesis and interpretation:** Main findings (e.g. interpretations, inferences, and themes); might include development of a theory or model, or integration with prior research or theory. | File: Manuscript_FINALv1  Pages: 9 to 21 |
| --- | --- |
| **Links to empirical data:** Evidence (e.g. quotes, field notes, text excerpts, photographs) to substantiate analytic findings. | File: Manuscript_FINALv1  Pages: 9 to 21 |

**Discussion**

| **Integration with prior work, implications, transferability, and contribution(s) to the field:** Short summary of main findings; explanation of how findings and conclusions connect to, support, elaborate on, or challenge conclusions of earlier scholarship, discussion of scope of application / generalizability; identification of unique contribution(s) to scholarship in a discipline or field. | File: Manuscript_FINALv1  Pages: 21-26 |
| --- | --- |
| **Limitations:** Trustworthiness and limitations of findings. | File: Manuscript_FINALv1  Page: 25 |

**Other**

| **Conflicts of interest:** Potential sources of influence or perceived influence on study conduct and conclusions; how these were managed. | N/A |
| --- | --- |
| **Funding:** Sources of funding and other support; role of funders in data collection, interpretation, and reporting. | Online submission |
